# Cephalopods’ Skin‐Inspired Design of Nanoscale Electronic Transport Layers for Adaptive Electrochromic Tuning

**DOI:** 10.1002/advs.202405444

**Published:** 2024-08-12

**Authors:** Yilin Yu, Xinyi Zhu, Shiqi Jiang, Shuangshuang Wu, Yu Zhao, Lingli Zhang, Liping Song, Youju Huang

**Affiliations:** ^1^ College of Material Chemistry and Chemical Engineering Key Laboratory of Organosilicon Chemistry and Material Technology Ministry of Education Hangzhou Normal University Hangzhou Zhejiang 311121 P. R. China

**Keywords:** adaptive electrochromic, camouflage skin, environment‐responsive, layer‐by‐layer assembly, nanoscale electronic transport

## Abstract

Cephalopods can change their skin color by using high‐speed electron transduction among receptors, neural networks, and pigmentary effectors. However, it remains challenging to realize a neuroelectrical transmission system like that found in cephalopods, where electrons/ions transmit on nanoscale, which is crucial for fast adaptive electrochromic tuning. Inspired by that, hereby an ideal, rapidly responsive, and multicolor electrochromic biomimetic skin is introduced. Specifically, the biomimetic skin comprises W_18_O_49_ nanowires (NWs) that are either colorless or blue, Au nanoparticles@polyaniline (Au NPs@PANI) ranging from green to pink, and a flexible conductive substrate. As the applied voltage changes from 0.4 V to ‐0.7 V and back to 0 V, the color of the biomimetic skin transforms from green to blue and ultimately to pink. This color change is attributed to the electrically induced redox reaction of Au NPs@PANI and W_18_O_49_ NWs, triggered by the transfer of electrons and ions. Furthermore, the high versatility and adaptability of electrical stimulus enable the creation of a highly interactive electrochromic biomimetic skin system through the integration of sensitive acoustic sensors, providing a perfect environment‐responsive platform. This work provides a biomimetic multicolor electrochromic skin that depends on electron/ion transfer on nanoscale, expands potential uses for camouflage skin.

## Graphical abstract

Here, a new design of nanoscale electronic transport layers by composing through the nanoscale growth of gold polymeric dots and the layer‐by‐layer assembly of nanowires and nanoparticles for adaptive electrochromic system is developed. Importantly, the highly controllable electrochromism at −0.7 V to 0.4 V has opened up opportunities to realize on‐demand camouflage skin.

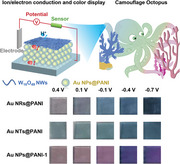

## 1 Introduction

Flexible intelligent color‐changing systems, embodying the attributes of flexibility,^[^ ^1^ ^]^ controllable stimulus‐response,^[^ ^2^ ^]^ and multicolor display capabilities,^[^ ^3^ ^]^ are highly significant in diverse fields such as smart sensing,^[^ ^4^ ^]^ information security,^[^ ^5^ ^]^ and dynamic camouflage.^[^ ^6^ ^]^ A prevalent approach involves integrating smart materials including photochromic,^[^ ^7^ ^]^ thermochromic,^[^ ^8^ ^]^ and electrochromic ^[^ ^9^ ^]^ materials, which are capable of altering their color into substrates.^[^ ^10^ ^]^ However, systems that rely on light or heat for color changes often involve intricate energy transfer processes, such as photovoltaic conversion and photothermal conversion,^[^ ^5b^ ^]^ which can lead to unwarranted energy consumption and sluggish switching speeds.^[^ ^11^ ^]^ Fortunately, these limitations can be effectively overcome by developing electrochromic systems.^[^ ^12^ ^]^ Zhao et al. designed a Janus‐structured two‐sided electrochromic device, which exhibits distinctly different coloration states on each side.^[^ ^13^ ^]^ Despite the numerous electrochromic systems reported, their applications in biomimetic skins remain limited. This might be attributed to the following reasons: 1) The electron/ion transfer process in electrochromic biomimetic skin is constrained by the limited contact area between the electrolyte and electrode, thereby significantly compromising both the response speed and cycling stability.^[^ ^14^ ^]^ 2) Although numerous electrochromic systems with wide color ranges and fast response times have been reported, they often lack environmental interactivity and intelligence. Therefore, exploring nanomaterials to enhance the contact area, and subsequently developing an intelligent system, is crucial and coincide with electrochromic material demand.

**Figure 1:**
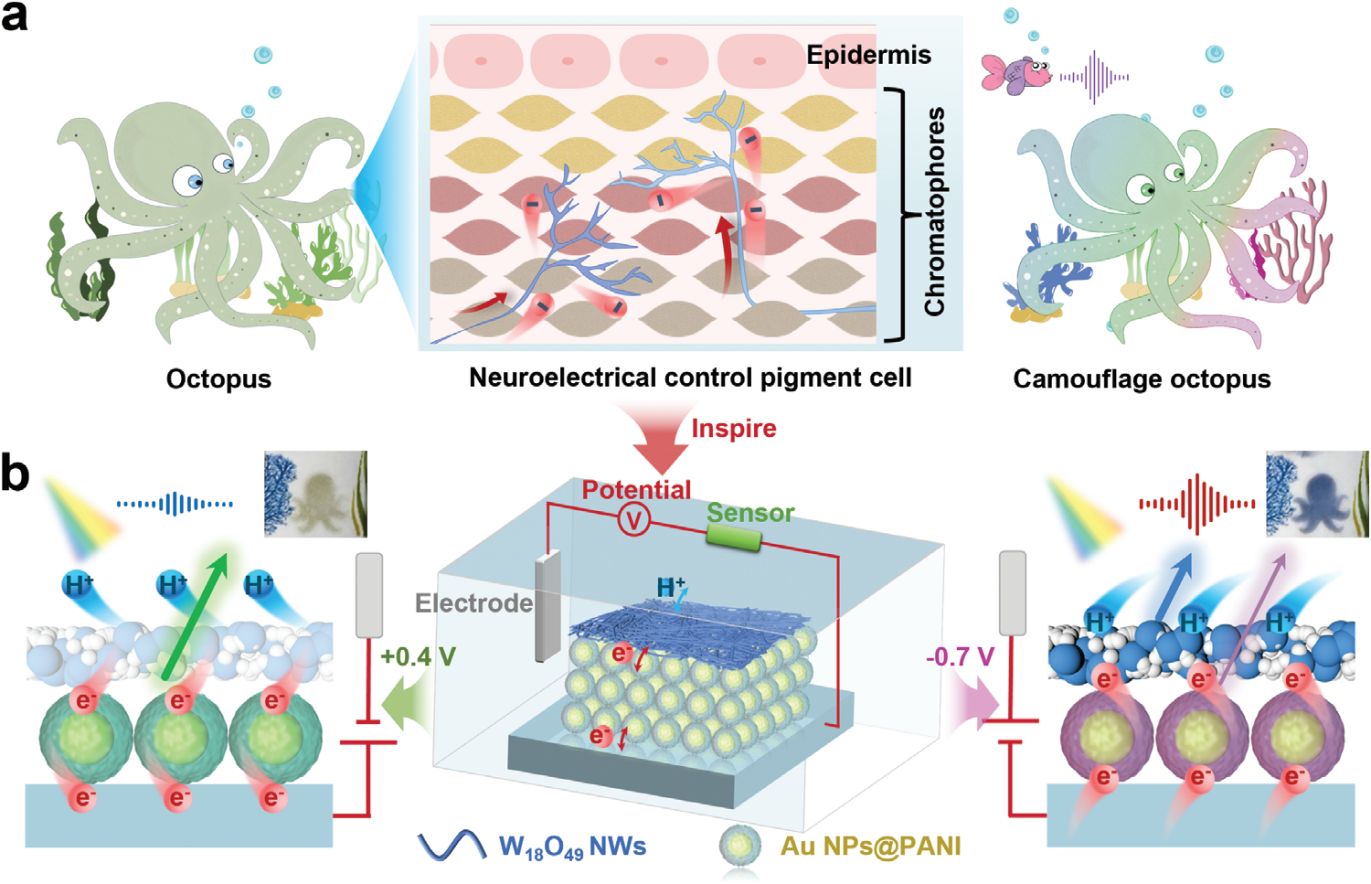
a) Schematic illustration for the dynamic color change of the octopus. Octopus dynamic camouflage relies on a neurologically regulated reflex arc, where sensory receptors in their visual and auditory systems assess surroundings, converting signals into electrical ones transmitted to the nervous system, ultimately controlling muscle cells around chromatophores to change skin color. b) Schemes illustrating the structure of the electrically controlled artificial chromatophore system, which combines sound sensors sensitive to environmental sound to automatically regulate the voltage on/off, intelligently inducing the electronic/ion transport of Au NPs@PANI and W_18_O_49_ NWs for color change.

The ability of certain living creatures to alter their skin color or body morphology by using high‐speed electron transduction among receptors hold a pivotal role in achieving self‐defense, camouflage, communication, and other essential biological processes. These fascinating natural phenomena have inspired researchers to design biomimetic structures and materials. Cephalopods are regarded as the most intelligent invertebrates, and their astounding structural color‐changing capabilities are attributed to the presence of various types of chromatophores and their intricately organized multilayer structures within their subcutaneous tissues though their neuroelectrical transmission system.^[^ ^15^ ^]^ As shown in **Figure** **1**a, octopus dynamic camouflage is facilitated through their neurologically regulated reflex arc. First, their visual and auditory system functions as sensory receptor, rapidly assessing complex environment and converting visual and sound signals into electrical impulses. Subsequently, these impulses are relayed through electronic/ionic conduction processes to the nervous system, which generates corresponding control commands. Finally, as the electronic/ionic signals to the skin surface, the muscle cells surrounding different chromatophore layers are actuated, leading to a change in skin color. Therefore, devising flexible electrochromic biomimetic skin, inspired by the skin structure and electronic/ionic transmission mechanisms of cephalopods, is an effective strategy to enhance their transmission rate and stimulus responsiveness.

In this article, we present a soft electrochromic biomimetic skin that employs layer‐by‐layer assembly of nanowires (NWs) and nanoparticles (NPs) to mimic the function of an artificial chromatophore. By harnessing nanoscale electronic/ionic transport mechanisms as neural electric signals, we trigger color modulation on demand. To design the nanoscale artificial chromatophore, we tightly couple the self‐assembly layer of Au NPs encapsulated in a conductive polyaniline shell (Au NPs@PANI) with W_18_O_49_ NWs,^[^ ^16^ ^]^ leveraging hydrophobic interaction forces between the two nanolayers. As shown in Figure 1b, when a voltage of −0.7 V was applied, electrons flow initially through the flexible substrate to the Au NPs@PANI layer, initiating a transition from green to pink. Almost simultaneously, the electrons proceed to the W_18_O_49_ NWs layer, promoting the migration of H^+^ ions from the surrounding solution into the NWs. This process leads to a change in color from colorless to blue, accompanied by a decrease in transparency. Ultimately, a superimposed color shift occurs within the nanoscale artificial chromatophore, transitioning it from green to blue. Conversely, when the voltage was switched to 0.4 V, the color reversed. Importantly, the versatility of colors exhibited by this artificial chromatophore can be fine‐tuned by adjusting the shape and size of Au used, the thickness of polyaniline and W_18_O_49_ NWs, and electrochemical parameter of the device. Furthermore, leveraging a thermal‐assisted planar nanostructure transfer printing technique, the electrochromic artificial chromatophore can be easily transferred to various conductive substrates, further demonstrating its remarkable flexibility. To further enhance its environmental responsiveness, sound sensors are integrated into the biomimetic skin, enabling it to dynamically alter its color in response to ambient sounds. This provides potential applications in the field of intelligent camouflage.

## 2 Results and Discussion

### 2.1 Design of the Artificial Chromatophore based on Nanoscale Growth of NPs and NWs

**Figure 2:**
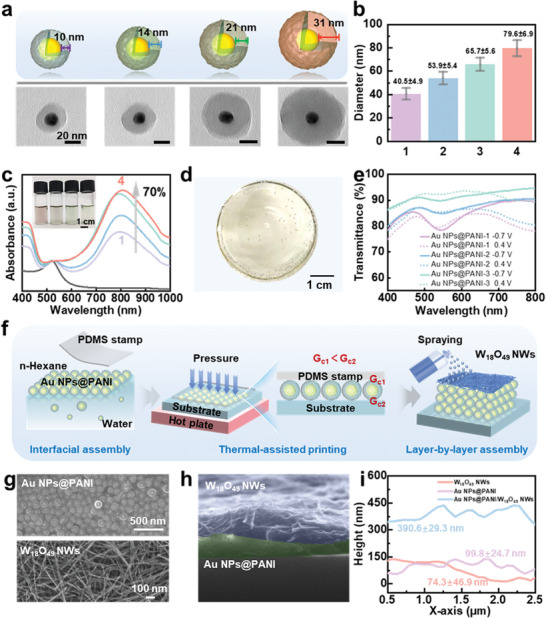
a) Schematic and transmission electron microscopy (TEM) images of Au NPs@PANI core/shell nanostructures with different shell thicknesses. The size of Au NPs@PANI increases gradually with prolonged polymerization time (from 12 h to 48 h). b) Diameter of Au NPs@PANI core/shell nanostructures with different shell thicknesses. c) The UV–vis absorbance spectra of Au NPs and Au NPs@PANI core/shell nanostructures with different PANI shell thicknesses. d) The optical photograph of AuNPs@PANI film at the liquid–vapor interface. e) UV–vis absorption spectra of AuNPs@PANI nanofilms (polymerized 12 h to 24 h) on ITO‐glass electrode at different applied potentials. f) Schematic illustration for co‐assembling Au NPs@PANI and W_18_O_49_ NWs. g) SEM images of Au NPs@PANI nanofilms and W_18_O_49_ NWs layer. h) Cross‐section SEM image of Au NPs@PANI/W_18_O_49_ NWs composite nanofilm. i) The height of Au NPs@PANI nanofilm, W_18_O_49_ NWs nanofilm and Au NPs@PANI/W_18_O_49_ NWs composite nanofilm.

To meet the requirements of the artificial chromatophore, some essential components should be included: an electroactive layer designed to facilitate transfer electrons and enable color switching, intermolecular hydrophobic interactions that create nano‐channels for expedited electron transport, and a proton transport layer with an adjustable absorption coefficient. The electroactive layer, capable of switching colors, is composed of self‐assembled Au NPs@PANI based on local surface plasmon resonance, and its color dynamics are achieved by altering the PANI molecular polarizability.^[^ ^14^ ^]^ First of all, the well‐defined Au NPs@PANI were synthesized through chemical oxidative polymerization process, following established methodologies reported previously.^[^ ^17^ ^]^ As shown in **Figure** **2**a–b and Figure S1, the PANI shell growth began at the interface and progresses radially outwards, resulting in the formation of Au NPs@PANI‐1, Au NPs@PANI‐2, Au NPs@PANI‐3, Au NPs@PANI‐4 with increasing polymerization time from 12 h to 48 h. The size of Au NPs@PANI‐1 to Au NPs@PANI‐4 increased gradually, ranging from approximately 40.5 ± 4.9 nm to 79.6 ± 6.9 nm. Meanwhile, the optical dynamics of polymerization were further evaluated by employing ultraviolet–visible (UV–vis) absorption spectroscopy. The results revealed a notable increase in absorbance intensity of approximately 70% as the thickness of the PANI shell expanded from roughly 10 nm to 31 nm. This increase in absorbance intensity was accompanied by a corresponding change in the color of the solutions, transitioning from red to green, as shown in Figure 2c. To ensure minimal optical near‐field coupling between the Au NPs@PANI and to implement a more abundant color range for Au NPs@PANI, two‐dimensional monolayer films of Au NPs@PANI were self‐assembled on an oil/water interface (Figure 2d).^[^ ^18^ ^]^ As shown in Figure 2e, the color of Au NPs@PANI (Au NPs@PANI‐1 to Au NPs@PANI‐3) nanofilms changed from red to green color, and its transmittance increased gradually. Once the power was turned on (−0.7 V), PANI^0^ is reduced to PANI^2−^ by accepting electrons from indium tin oxide (ITO)‐glass electrode, and the transmittance band of 700 nm to 800 nm suffered a significant increase. Then, the Au NPs@PANI‐3 nanofilms were chosen to construct the artificial chromatophore systems owing to their capability to exhibit a diverse range of color changes spanning from green to red.

The artificial chromatophore was fabricated using a layer‐by‐layer composite methodology. As shown in Figure 2f, the self‐assembled Au NPs@PANI nanofilms were transferred from the oil/water interface onto a conductive substrate via thermal‐assisted printing technology employing a polydimethylsiloxane (PDMS) stamp. During the printing process, two crucial points should be considered. Firstly, the adhesion energy between the PDMS stamp and the nanofilms (**G_c1_**) should be weaker than the adhesion energy between the nanofilms and the receiving substrate (**G_c2_**).^[^ ^19^ ^]^ Therefore, the hydrophilic treatment of the receiving substrate is indispensable. Secondly, thermal assistance is essential for the printing technology.^[^ ^20^ ^]^ The success rate of printing increased significantly at 45 °C and then remained stable (Figure S2). At this temperature, Au NPs@PANI multilayer nanofilms were prepared by repeating the thermal‐assisted printing process. Subsequently, the W_18_O_49_ NWs layer was tightly adhered to the Au NPs@PANI nanofilms through spraying the W_18_O_49_ NWs solution (Figure S3). As shown in Figure 2g, the Au NPs@PANI layer serves as a stable and conductive intermediary between the W_18_O_49_ NWs layer and the conductive substrate. Its robust bonding to both layers is attributed to two primary factors: hydrophobic effects and hydrogen bonding interactions. The hydrophobic surfaces of both the Au NPs@PANI layer and the conductive substrate promote strong interfacial affinity, while hydrogen bonding between the ‐N‐H groups of the polyaniline in the Au NPs@PANI layer and the ‐O‐ of the W_18_O_49_ NWs ensures stable adhesion. Furthermore, the Au NPs@PANI layer possesses excellent conductivity, facilitating efficient electron transport across the interface. These properties collectively contribute to the stable and efficient functioning of the overall device structure. The thickness and color of the W_18_O_49_ NWs layer can be precisely regulated by varying the volume of the sprayed solution, varying from colorless to light blue (Figure S4). The rapid electron transport ability between the layers of the as‐prepared artificial chromatophore was validated through scanning electron microscopy (SEM) and atomic force microscopy (AFM). As shown in Figure 2h, the tri‐layer interfacial microstructure of the artificial chromatophore exhibited a tight, gap‐free integration. Additionally, the AFM results indicate that, with the process of layer‐by‐layer self‐assembly, the thickness increased from ∼74.3 ± 49 nm to ∼390.6 ± 29 nm (Figure 2i).

### 2.2 Electrochromic Artificial Chromatophore Modulated by Nanoscale Electronic Transport

**Figure 3:**
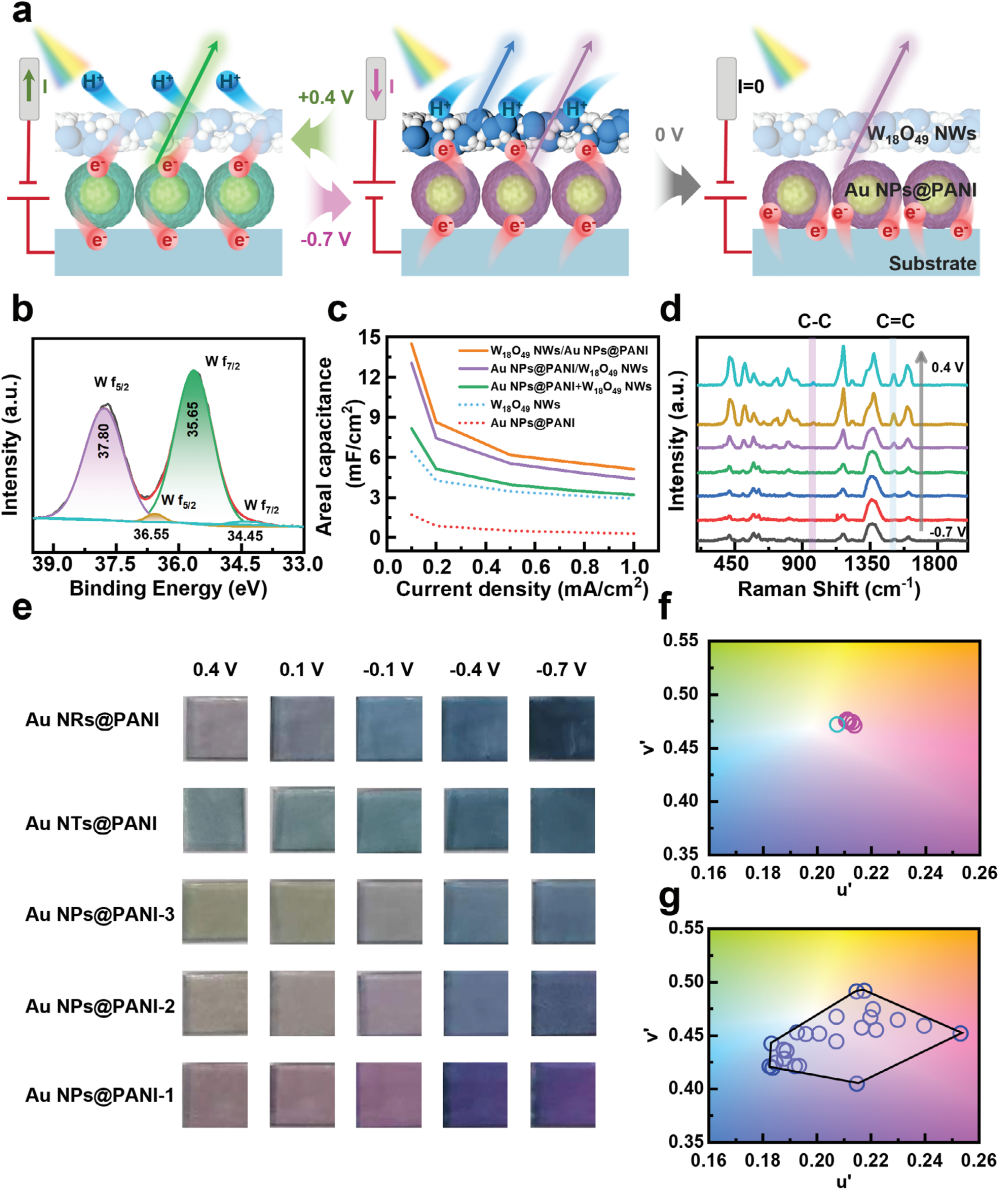
a) Schematic illustration of the movement of H^+^ and e^−^ during the electrochromic process under different bias voltage. b) High‐resolution XPS spectra for W 4f of Au NPs@PANI/W_18_O_49_ NWs composite nanofilm. c) The areal capacitance of the Au NPs@PANI, W_18_O_49_ NWs, Au NPs@PANI/W_18_O_49_ NWs, and W_18_O_49_ NWs/Au NPs@PANI nanofilms at different current densities of 1.0, 0.8, 0.5, 0.2, and 0.1 mA cm^–2^. d) Raman spectra of Au NPs@PANI/W_18_O_49_ NWs composite nanofilm at different voltages. e) Optical pictures of the three‐layered nanofilm structure, which comprises Au NPs@PANI‐1, Au NPs@PANI‐2, Au NPs@PANI‐3, Au NRs@PANI, and Au NTs@PANI, with W_18_O_49_ NWs layer of different thickness, captured at potentials ranging from 0.4 to −0.7 V. f) Enlarged CIE color coordinates of Au NPs@PANI and W_18_O_49_ NWs at different applied potentials, along with photographs of corresponding samples in Figures S8. g) Enlarged CIE color coordinates of different composite nanofilm at different applied potentials, along with photographs of corresponding samples in (e).

The overlapping color‐changing kinetics of the as‐prepared artificial chromatophore is illustrated in **Figure** **3**a. Upon applying a voltage of −0.7 V, a prominent overlapping color shift was observed, transforming the artificial chromatophore from green to blue. This color‐tunable phenomenon can be explained as follows: upon voltage‐induced electron transfer, the Au NPs@PANI layer underwent a reduction process, transforming PANI from its oxidized state (Au NPs@PANI^0^, green) into its reduced state (Au NPs@PANI^2−^, red). Concurrently, the colorless W_18_O_49_ NWs layer converted to the blue‐colored H_x_W_18_O_49_ by receiving electrons from the Au NPs@PANI layer and protons (H^+^) from the electrolyte. This donor (PANI)‐acceptor (W_18_O_49_) pair functioned as a nanoscale electronic transport tunnel, further enhancing the electron/proton transfer process of them.^[^ ^21^ ^]^ Intriguingly, upon removal of the −0.7 V voltage, the bright blue color of the artificial chromatophore turned pink firstly and then gradually reverted to green in a sequential manner. This color transition was attributed to the fact that H_x_W_18_O_49_ (blue colored) converts to W_18_O_49_ (colorless) at a higher rate, while Au NPs@PANI^2−^ (red) remained constant initially and then gradually recovered to its oxidized state, Au NPs@PANI^0^ (green) at a lower rate. In contrast, when a reverse electric field (0.4 V) was applied, the bright blue color of the artificial chromatophore immediately recovered to green, as the H_x_W_18_O_49_ (blue colored) converts to W_18_O_49_ (colorless) and Au NPs@PANI^2−^ (red) transforms into Au NPs@PANI^0^ (green) simultaneously. To study the chemical and electronic states of Au NPs@PANI and W_18_O_49_ NWs, X‐ray photoelectron spectroscopy (XPS) is employed. The analysis of the results showed characteristic peaks of wolfram (W) in the Au NPs@PANI/W_18_O_49_ NWs composite nanofilm, corresponding to the W (4f7/2) and W (4f5/2) core levels of W^6+^ (peaks at 35.65 eV and 37.80 eV) and W^5+^ cations (peaks at 34.45 eV and 36.55 eV) (Figure 3b), implied the mixed valence of W. Importantly, after layer‐by‐layer assembly, the binding energy of nitrogen (N) can be found slightly enhanced in the XPS spectrum of the Au NPs@PANI/W_18_O_49_ NWs composite nanofilm than that of pure Au NPs@PANI, which indicated N atoms lose electrons when the W atom gained electrons in the artificial chromatophore (Figure S5). Furthermore, the artificial chromatophore exhibited a higher areal capacitance than both pure Au NPs@PANI and W_18_O_49_ NWs nanofilms at different current densities. This significant improvement in the effective dielectric constant was due to the interaction between the two electrochromic materials (Figure 3c and Figure S6).

### 2.3 Electrically Controlled Color‐Changing Process

The electrically controlled color‐changing processes of the artificial chromatophore which consists of three layers of Au NPs@PANI and W_18_O_49_ NWs nanofilm, were thoroughly studied. As shown in Figure S7, upon applying a voltage of −0.7 V, the artificial chromatophore displayed a bright blue color. Subsequently, by gradually increasing the voltage from −0.7 V to −0.3 V, and then to 0.5 V, the color shifted from blue to purple and ultimately transformed into green UV–vis absorption spectroscopy revealed that the transmission peak, originally at 400 nm, gradually red‐shifted to 650–700 nm as the voltage increased from −0.7 V to −0.3 V, resulting in a superimposed purple hue of the artificial chromatophore. Upon reaching a voltage of 0.5 V, the transmittance peak shifted to around 570 nm, resulting in the appearance of green color. This color change was attributed to the varying degrees of reduction reaction and hybridization that occur between PANI and W_18_O_49_ NWs. To further delve into the electrochromic mechanism of the artificial chromatophore, surface‐enhanced Raman spectroscopy was continuously monitored under varying voltages (Figure 3d). All Raman characteristic bands of the artificial chromatophore increased as the voltage increased, attributed to the improvement of molecular polarizabilities. The Raman characteristic peaks of C‐C (around 990 nm) and C = C (around 1598 nm) increased notably, implying the redox reaction of PANI. Furthermore, the artificial chromatophores with a variety of colors could be further prepared by treating the composite nanofilm in mixtures of Au NPs@PANI/W_18_O_49_ NWs with different ratios. With an increase in diameter from 40.5 ± 4.9 nm to 65.7 ± 5.6 nm, the color of Au NPs@PANI (both layers self‐assembled) gradually turned from red to green color, whereas W_18_O_49_ NWs remained colorless (Figure S8). However, when a voltage of −0.7 V was applied, only a slight change in color was observed for the composite film, with the Commission Internationale de L’ Eclairage 1976 chromaticity diagram (CIE) coordinates of (0.2074, 0.4720), (0.2137, 0.4709) and (0.2111, 0.4765) (Figure 3f). Once the Au NPs@PANI and W_18_O_49_ NWs were assembled, the artificial chromatophore showed a marked contrast under different applied voltages, and a much larger range of colors was visible with increasing thicknesses of the W_18_O_49_ NWs layer (from 11.6 ± 7.8 nm to 145.3 ± 28.2 nm), namely, hermosa pink, light purple, indigo blue, tawny, light gray, and gray (Figure S9, S10). Remarkably, the color palette of the artificial chromatophore can be significantly broadened by fine‐tuning the morphology (shape and size) of the Au, the thickness of the polyaniline coating, and the electrochemical parameters of the device. As demonstrated in Figures S11–S13, by transforming the shape of the Au from nanorods (NRs) to nanotriangles (NTs), and subsequently to nanobipyramids (NBPs), the absorption band of the PANI‐coated Au extends across the entire visible spectrum. Consequently, the artificial chromatophore composed of Au NPs, NRs, and NTs exhibits a diverse color transition ranging from red, through orange, yellow, green, to violet (Figure 3e). Additionally, as the applied voltage varies from 0.4 V to −0.7 V, the corresponding CIE coordinates shift from (0.2534, 0.4512) to (0.2149, 0.4041), (0.2178, 0.4913), and finally to (0.1833, 0.4415), as shown in Figures 3e,g. In addition, a 3 × 3 square array of the artificial chromatophore with different combinations of Au NPs@PANI and W_18_O_49_ NWs nanofilms was constructed to demonstrate its high color tunability (Figure S14). Thus, the artificial chromatophore with tunable multicolor is expected to be a novel kind of smart electrochromic material promising for various applications.

### 2.4 Patterned Display and Practicality Studies

**Figure 4:**
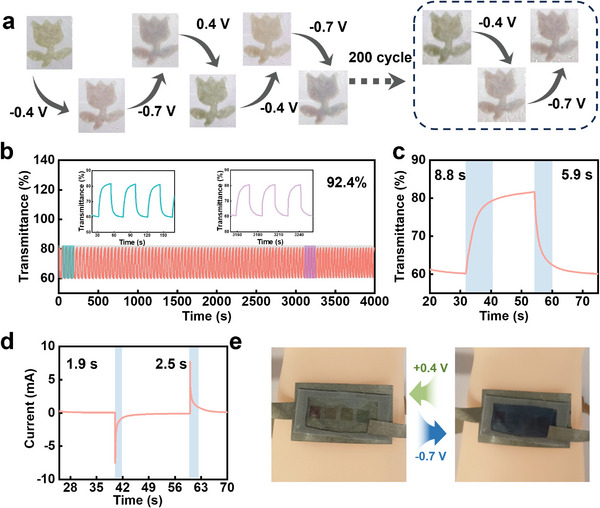
a) Optical picture of Au NPs@PANI/W_18_O_49_ NWs composite nanofilm at a voltage of −0.7, −0.4, and 0.4 V for 10 s for cycles. b) Electrochromic switching behaviors of the Au NPs@PANI/W_18_O_49_ NWs composite nanofilm monitored at 600 nm at a voltage of −0.7 V for 20 s and 0.4 V for 20 s for cycles. c) Electrochromic switching behaviors of the Au NPs@PANI/W_18_O_49_ NWs composite nanofilm monitored at 600 nm at a voltage of −0.7 V for 30 s and 0.4 V for 30 s for cycles. d) The chronoamperometry (CA) curves of the Au NPs@PANI/W_18_O_49_ NWs composite nanofilm at the alternant potential of −0.7 and 0.4 V. e) Optical picture of the device consisting of nickel fabric and Au NPs@PANI/W_18_O_49_ NWs composite nanofilm at 0.4 and −0.7 V.

In demonstrate the feasibility of utilizing the electrically controlled color of the artificial chromatophore, different patterns were constructed. As shown in **Figure** **4**a, a colorful flower was designed, which switches deftly between variable colors at different voltage levels. The color‐changing processes are displayed for three distinct stages, revealing a clear color transformation of the flower from green to pink and then to purple as the voltage is altered (from 0.4 V, to −0.4 V and then to −0.7 V). This process was reversible and could be repeated for 200 times upon switching of the voltage. The longer durability was measured by providing a continuous voltage pulse to the artificial chromatophore for a sufficiently long period of time and observing its optical response. Figure 4b demonstrates an example of the artificial chromatophore, exhibiting a stable performance up to 4000 s with minimal loss in transmittance values (lower than 8%), which indicates its excellent stability performance. Furthermore, the combination of Au NPs@PANI and W_18_O_49_ NWs significantly boosts the optical switching capability of the artificial chromatophore, which is described by the improved switching time. The coloring or bleaching time was defined as the time taken for the artificial chromatophore to reach 90% of its maximum transmittance. As illustrated in Figure 4c and S15,S16, owing to the excellent electronic transmission capability of the nano tunnels, the switching time of the artificial chromatophore (coloring time ∼ 5.9 s, bleaching time ∼ 8.8 s), providing a promising foundation for practical applications. To help quickly and systematically evaluate the performance of the artificial chromatophore, some generally used and crucial electrochromic performance indexes including optical modulation (∆*T*), contrast ratio (CR), response speed (*v*) and coloration efficiency (CE) were further calculated (see in the experimental section). Accordingly, the ∆*T* and CR of the artificial chromatophore were about 21.48% and 1.36, which proves the high contrast of color. Moreover, the *v* and CE were calculated about 0.0219 s^−1^ and 19.84 cm^2^ C^−1^ (Figure S18) to quantify the processes of optical modulation, which demonstrated the well energy efficiencies of the artificial chromatophore. Not only that, the artificial chromatophore supported a fastcurrent response of less than 3 s, as shown in Figure 4d and S17. It is noteworthy that the difference in response time stems from the inherent disparity in ion and electron kinetics during the typical color‐changing process of W_18_O_49_ NWs. Specifically, the speed of ion transport is notably slower than that of electron transport, leading to a delay in the color change as compared to the corresponding change in current as previously reported.^[^ ^22^ ^]^ In addition, a multi‐colored chameleon comprising three distinct sectors was crafted using varying compositions of composite nanofilms (consisting of three layers of Au NPs@PANI‐1, Au NPs@PANI‐2, Au NPs@PANI‐3 with the same W_18_O_49_ NWs, from left to right), exhibiting a differentiated color display (Figure S19). Thanks to the versatility of thermal‐assisted printing technology, Au NPs@PANI/W_18_O_49_ NWs can be transferred onto various interfaces with different roughness levels, such as nickel fabric, ITO film, and copper tape. For instance, when coated on nickel fabric, the Au NPs@PANI/W_18_O_49_ NWs composite exhibits remarkable flexibility and interface stability, allowing it to be easily folded, bent, and twisted multiple times without damage (Figure 4e). In addition, it was found that the flexible devices exhibit stable color‐changing performance while it was affixed to an arm model. These results indicate the quick electronic/ion transfer rate of the artificial chromatophore, which is crucial for achieving elegant control of electrically controlled color‐changing behaviors.

### 2.5 Application as Biomimetic Skins

**Figure 5:**
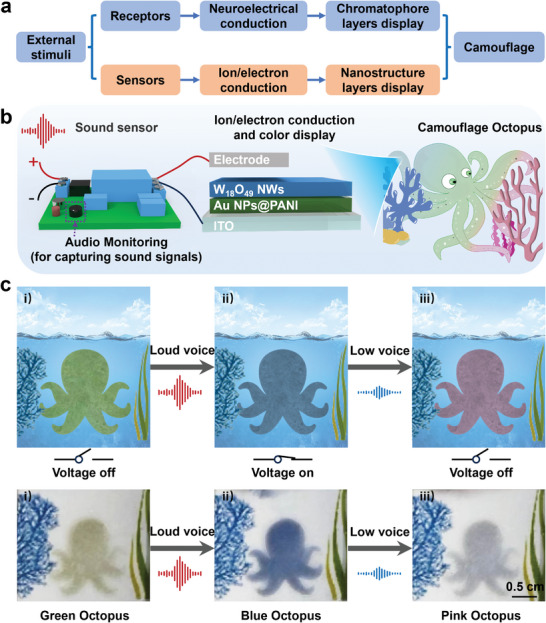
a) Flow chart of stimulation response, signal transduction and skin camouflage of octopus and biomimetic device in the face of environmental change. b) Illustration showing the multilayered structure of artificial chromatophore, as well as bionic octopuses, sound sensor, and battery into electrochromic biomimetic system. c) Schematic and optical images of octopus discoloration in different environments. i) Green bionic skins floated in water. ii) The ambient sound gradually increases, the sensor automatically triggers the connection of the circuit, the color of bionic skins changes to blue. iii) As the sound diminishes, the sensor automatically disconnects the circuit, the bionic skins turn to pink.

It is well‐known that developing suitable bionic skins is worthwhile to assist robots in blending into their backgrounds (camouflage) or standing out against them (warning) in environments.^[^ ^23^ ^]^ For instance, camouflaged robots can hide and protect themselves in various environments, making it easy for them to carry out reconnaissance tasks. The octopus, as a marine creature, possesses an intricate and functionally complex body structure, with its response mechanism to external environmental stimuli being a marvelous feat in the biological world. As shown in **Figure** **5**a, when confronted with external stimuli, such as abnormal sounds, the octopus’ sensory organs rapidly capture and convert these stimuli into electrical signals. Subsequently, these signals were rapidly transduced and processed through its highly developed nervous system, enabling it to assess potential threats and make reactive decisions. Once the need for camouflage arises, the nervous system dispatches instructions to effectors like chromatophores, allowing the octopus to swiftly transform from its original color to a camouflage hue, achieving seamless integration with its surroundings. This showcases the octopus’ unique survival wisdom and biological adaptability. Inspired by octopus’ skin and leveraging the aforementioned excellent voltage‐controlled diverse color‐changing characteristics, this artificial chromatophore was subsequently employed as a bionic skin.

To achieve the design of a stimulus‐responsive intelligent color‐changing skin, we ingeniously introduced an acoustic sensor capable of automatically connecting circuits through sound signals (Figure S20). This design inspiration originated from the keen perception and response mechanisms of organisms to external stimuli in nature, aiming to mimic the intelligent color‐changing behavior of biological bodies in complex environments. As shown in Figure 5b–c and Movie S1, we carefully crafted bionic skins, which were connected to a power source and a sound switch, respectively, and placed in a clear aqueous solution (0.5 M NaCl, 0.01 M HCl). When the ambient sound level gradually increases, for instances, in response to the sound of an enemy in a realistic scenario, the sound sensor in the system acts like a sensitive receptor of the bionic skins, quickly capturing these sound signals. Once the sound signals exceed a predefined threshold, the sensor automatically initiates the circuit connection, triggering the exchange of electrons and protons between nanospheres and nanowires. This process results in the color of the bionic skins changing from its original green to a blue camouflage skin that better blends with the surrounding environment. Remarkably, when the external sound gradually weakens, the sensor is equally capable of perceiving this change and automatically disconnecting the circuit. As the circuit disconnects, the camouflage skin gradually shifts from blue to pink. This color shift aids the bionic skin in blending into its surroundings more effectively but also functions as a means to attract prey, thus enhancing its hunting capabilities. This dynamic color‐changing process is highly similar to the real‐life mechanism of octopus color changes, successfully achieving the intelligent interaction between the electrochromic skin and its environment. It fully demonstrates the tremendous potential of biomimetics in the design of intelligent materials. By mimicking the intelligent behaviors of biological organisms, we are hopeful of developing more intelligent materials with practical applications, paving a new path for future technological advancements.

## 3 Conclusion

This article delves into a groundbreaking and innovative soft electrochromic bionic skin that meticulously mimics the complex and remarkable skin structure of cephalopods, particularly octopuses. This bionic skin not only resembles real biological skin in appearance but also achieves rapid and reversible color changes in functionality, a feature attributed to its unique electron/ion transport mechanism. To construct this artificial chromatophore, advanced layer‐by‐layer assembly techniques were employed, precisely combining Au NPs@PANI and W_18_O_49_ NWs. This nanoscale material design enables the bionic skin to rapidly respond to voltage changes, exhibiting a diverse array of colors. As the voltage varies, the flow of electrons and ions within the nanostructure triggers rapid color changes, transforming the skin from green to blue. Notably, the diversity of colors can be further enriched by adjusting the thickness and composition of the nanomaterials, creating more diversified camouflage effects. This flexible color‐changing characteristic positions the bionic skin as a promising candidate in areas such as military camouflage, biomimicry, and intelligent material design. To enhance the intelligence of the bionic skin, acoustic sensors were ingeniously integrated into the design, further mimicking the cephalopods’ sensitive response to environmental stimuli. When external sound signals alter, the acoustic sensors rapidly capture and convert them into electrical signals, triggering color changes in the bionic skin. Although the responsive color changes system's practical application is limited by its immature performance, but future improvements in system miniaturization, integration with advanced electronics, sensor sensitivity, and wireless communication could enhance its practicality and versatility. Most importantly, this intelligent response mechanism allows the bionic skin to react accordingly to environmental changes, further strengthening its application value in the design of intelligent materials.

## 4 Experimental Section

### Materials

Chloroauric acid (HAuCl_4_•3H_2_O, 99.9%) and sodium citrate (SC, ≥99.0%) were purchased from Sigma‐Aldrich. Poly(vinylpyrrolidone) (PVP, MW ≈ 40 000) and Hexane (≥97.0%) were bought from Energy Chemical. Sodium dodecyl sulfate (SDS, ≥99.0%), aniline (≥99.0%), ammonium persulfate ((NH_4_)_2_S_2_O_8_, ≥98%), 1H‐1H‐2H‐2H‐perfluorodecanethiol (PFT, 97%) and tungsten chloride (WCl_6_, 99.9%) were obtained from Aladdin. Sodium chloride (NaCl, ≥99.8%), hydrochloric acid (HCl, 37 wt% in water) and ethanol (99.7%) were purchased from Sinopharm Chemical Reagent Co., Ltd. Polydime‐thylsiloxane (PDMS, DOW 184) was purchased from Shanghai Sadhu Trading Co. All chemicals were used without further purification.

### Synthesis of Au NPs

Au NPs with a diameter of 15 nm were synthesized according to a reported method.^[^ ^24^ ^]^ Briefly, HAuCl_4_ (1 mL, 25 mM) was added to deionized water (96 mL) and set temperature to 110 °C. Then, SC (1 mL, 0.1 M) was added with the temperature of the oil bath maintained at 110 °C for 30 min. Keep stirring until cooled to room temperature.

### Synthesis of Au NPs@PANI Core/Shell Nanostructures

Au NPs@PANI was synthesized according to our reported method.^[^ ^17^ ^]^ The as‐synthesized Au NPs (45 mL) were concentrated at 12 000 rpm for 10 min to remove the supernatant. Then, the precipitate was redispersed in a mixture of aniline (6 µL) and SDS (7.5 mL, 40 mM). Then, the solution was ultrasonic concussion for 1 min. The resultant solution was mixed with an acidic (NH_4_)_2_S_2_O_8_ solution (45 mL, 2 mM in 10 mM HCl) and ultrasonic for 30 s. Next, the mixture was incubated at room temperature for 12 h. Finally, the free PANI is removed by centrifugation at 12 000 rpm. Repeat the previous steps to obtain a series of core/shell structures with different shell thicknesses.

### Fabrication of Au NPs@PANI Nanofilm

The Au NPs@PANI nanofilm was prepared according to our previous method with some modifcations.^[^ ^18^, ^25^ ^]^ Briefly, the Au NPs@PANI (10 mL) were added into a glass beaker (4.5 cm in diameter), and PFT (10 mL, 10 mM V_ethanol_:V_hexane_ = 1:2) was added to the Au NPs@PANI solution, to obtain Au NPs@PANI nanofilm.

### Fabrication of PDMS

PDMS prepolymer and curing agent were mixed at a weight ratio of 10:1. The mixture was thoroughly stirred, and poured into a plastic petri dish. PDMS mixture was standing until all bubbles were removed, and then cured in the oven at 70 °C for 2 h.

### Transfer of Au NPs@PANI Nanofilm

The PDMS was attached to Au NPs@PANI by the standard adhesion method, and covered on the plasma‐treated indium tin oxide (ITO) glass. Then, please it in the oven at 80 °C for 10 min to transfer Au NPs@PANI to ITO glass.

### Synthesis of W_18_O_49_ NWs

Uniform W_18_O_49_ NWs were prepared by a modified solvothermal method.^[^ ^16^ ^]^ Typically, 0.0001 g of PVP and 0.03 g of WCl_6_ were added into 40 mL of ethanol. The mixture was stirred by magnetic stirring for 10 min. The solution was then added into a 50 mL Teflon‐lined stainless‐steel autoclave and maintained at 200 °C for 12 h. After that, the autoclave was cooled to room temperature naturally and W_18_O_49_ NWs were obtained. Finally, the prepared W_18_O_49_ NWs solution was centrifuged and washed in absolute ethanol for several times, then redissolve into ethanol (10 mL).

### Fabrication of W_18_O_49_ NWs Nanofilm

Take a certain volume of W_18_O_49_ NWs solution, followed by ultrasonication for 3 min to obtain homogeneous solution. In the spray‐coating process, the distance between the ITO glass and the airbrush gun was fixed at about 10 cm, while place the plasma‐treated ITO glass under infrared ovens lamp to accelerate the solvent evaporation.

### Calculation of basic Electrochromic Data

It was found that the optical modulation (∆*T*) and contrast ratio (CR) of the artificial chromatophore were about 21.48% and 1.36, according to Equation 1 and Equation 2.^[^ ^26^ ^]^

(1) $$\Delta T=T_{\mathrm{bleached}}-T_{\mathrm{colored}}$$

(2) $$CR=T_{\mathrm{bleached}}/T_{\mathrm{colored}}$$

Here, *T*_bleached_ and *T*_colored_ represent the transmittance in the bleached state and colored stat, respectively.

On the other hand, the response speed (*v*) and coloration efficiency (CE) were calculated about 0.0219 s^−1^ and 19.84 cm^2^ C^−1^ to quantify the processes of optical modulation, according to Equation 3 and Equation 4,Equation 5.^[^ ^6c^ ^]^

(3) v=ΔT/t

(4) $$\Delta \mathrm{OD}=\log\left(T_{\mathrm{bleached}}/T_{\mathrm{colored}}\right)$$

(5) CE=ΔOD/ΔQ

### Device Characterization

The substrate morphology was examined by transmission electron microscopy (TEM; HT‐7700, Hi‐tachi, Japan) and scanning electron microscopy (SEM; Supra5, Zeiss, Germany). The X‐ray photoelectron spectra (XPS) was recorded on a Thermofisher ESCALAB Xi+ X‐ray photoelectron spectrometer (Thermo, America). Atomic force microscope (AFM) was recorded on a Dimension ICON atomic force microscope (Bruker, America). The optical proper‐ties of the prepared materials were examined by ultraviolet‐visible (UV–vis) spectroscopy (Purkinje General Instrument Co., Ltd., Beijing, China). Electrochemical measurements were carried out using a three electrodes system on an LC‐LX‐H165A (LiChen, China). Ultrapure water was produced by Milli‐Q ultrapure water system (Millipore, Bedford, USA).

## Conflict of Interest

The authors declare no conflict of interest.

## Supporting information

Supporting Information

Supplemental Movie 1

## Data Availability

The data that support the findings of this study are available in the supplementary material of this article.
