# Supplementary material for: Cephalopods’ Skin‐Inspired Design of Nanoscale Electronic Transport Layers for Adaptive Electrochromic Tuning
Source: Adv Sci (Weinh). 2024 Aug 12;11(39):2405444. doi: 10.1002/advs.202405444 (PMC11633331; doi:10.1002/advs.202405444)
Supplement: Supplementary file 1 — Supporting Information [file ADVS-11-2405444-s001.docx]

Supporting Information

**Cephalopods’ Skin-Inspired Design of Nanoscale Electronic Transport Layers for Adaptive Electrochromic Tuning**

Yilin Yu, Xinyi Zhu, Shiqi Jiang, Shuangshuang Wu,* Yu Zhao, Lingli Zhang, Liping Song,* Youju Huang*


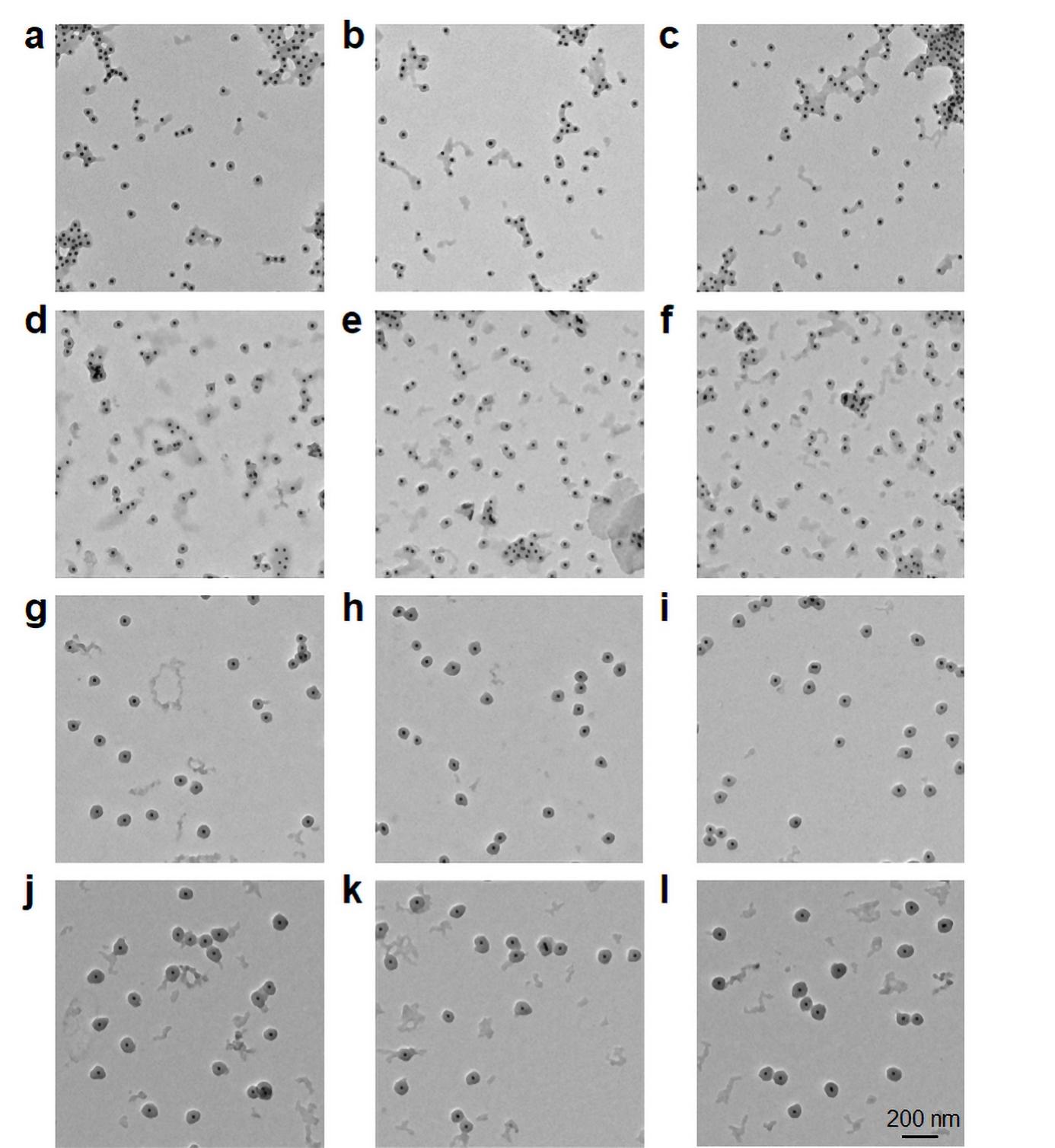


**Figure S1****.** TEM images of Au NPs@PANI-1 (a-c), Au NPs@PANI-2 (d-f), Au NPs@PANI-3 (g-i) and Au NPs@PANI-4 (j-l). The sizes of Au NPs@PANI-1 to Au NPs@PANI-4 are 40.5 ± 4.9, 53.9 ± 5.4, 65.7 ± 5.6 and 79.6 ± 6.9 nm.


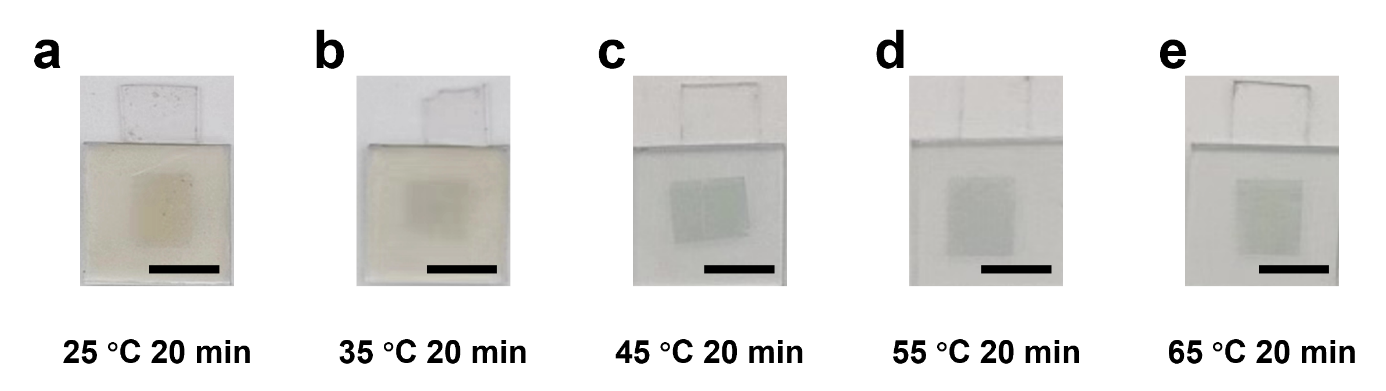


**Figure S2.** (a-e) Optical pictures of transferability Au NPs@PANI nanofilms at different temperatures for 20 min. Before the temperature reaches 45 ^o^C degrees, there is some loss of printing. Scale bar: 0.5 cm.


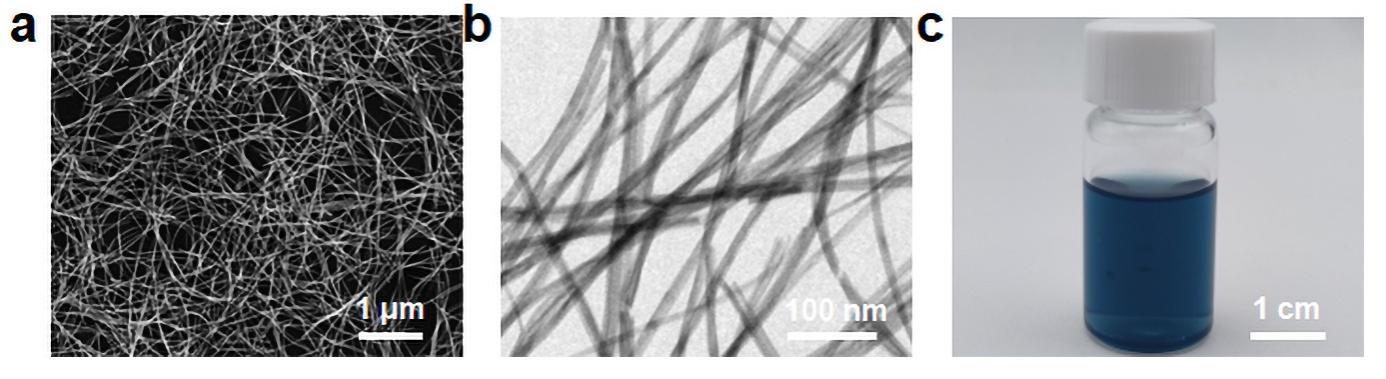


**Figure S3.** (a-b) SEM and TEM images of W_18_O_49_ NWs. (c) Optical picture of W_18_O_49_ NWs solution (0.0008 g/ml).


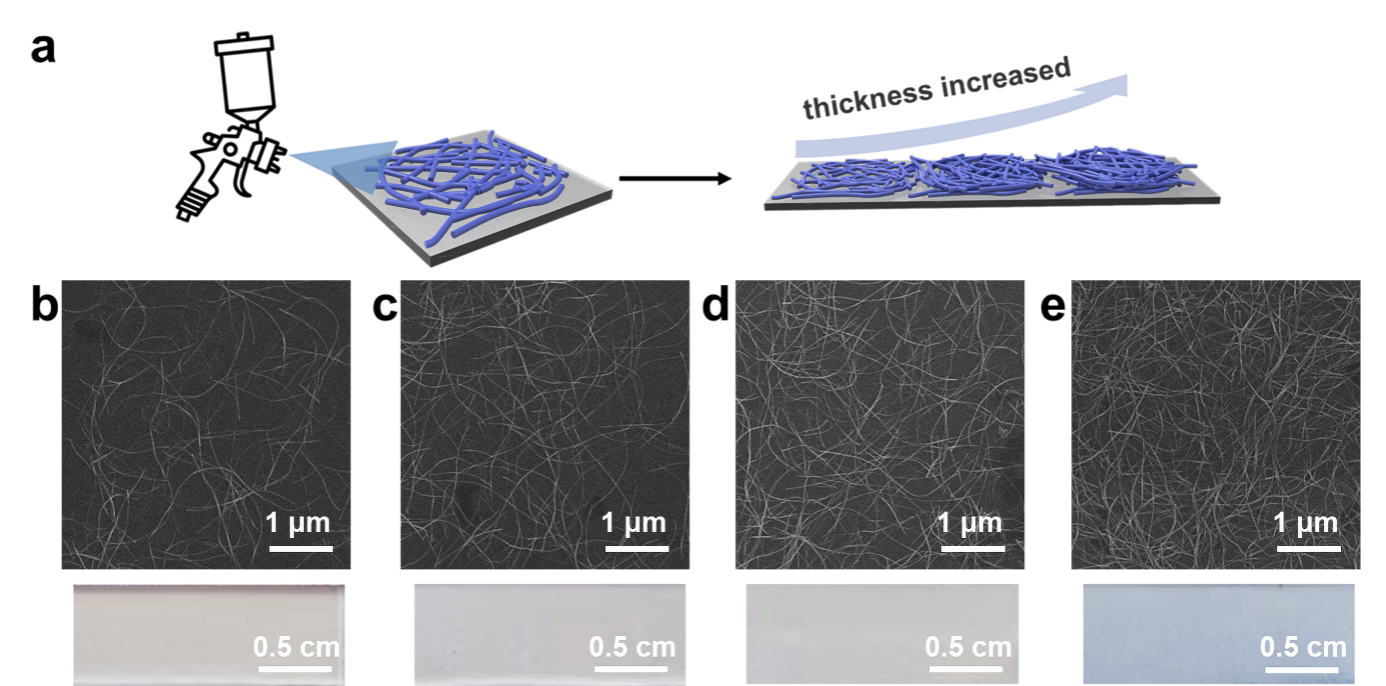


**Figure S4**. (a) Scheme of W_18_O_49_ NWs layer construction. (b-e) SEM images and optical pictures of different volume (0.05, 0.1, 0.15 and 0.2 mL) of W_18_O_49_ NWs at -0.7 V.


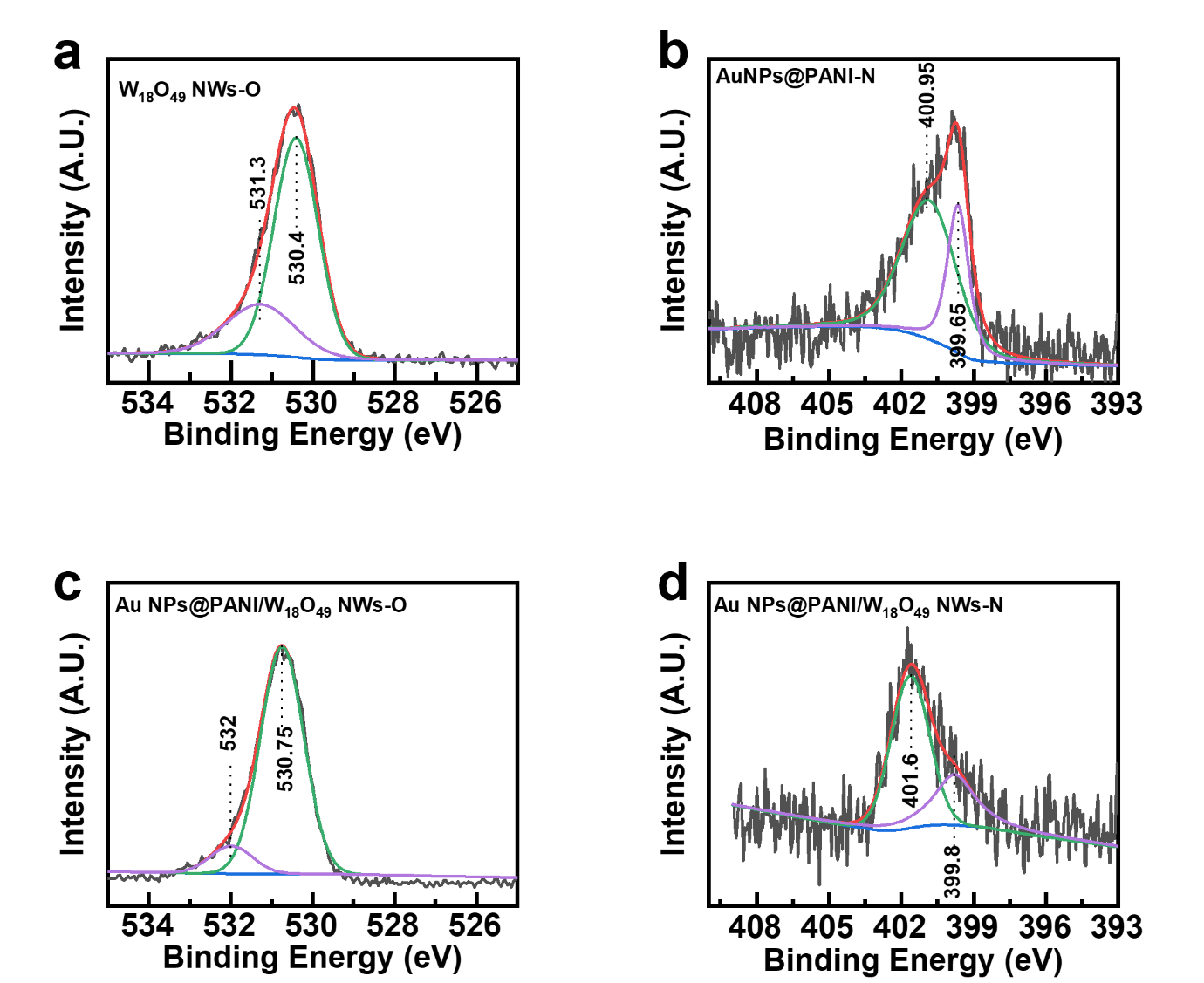


**Figure S5.** High-resolution XPS spectra for (a) O 1s of pure W_18_O_49_ NWs. (b) N 1s of pure AuNPs@PANI. (c) O 1s and (d) N 1s of the mixture of AuNPs@PANI/W_18_O_49_ NWs. After layer-by-layer assembly, the binding energy of nitrogen (N) can be found slightly enhanced in the XPS spectrum of the Au NPs@PANI/W_18_O_49_ NWs composite nanofilm than that of pure Au NPs@PANI, which indicates N atoms loose electrons. At the same time, W atom gains electrons.


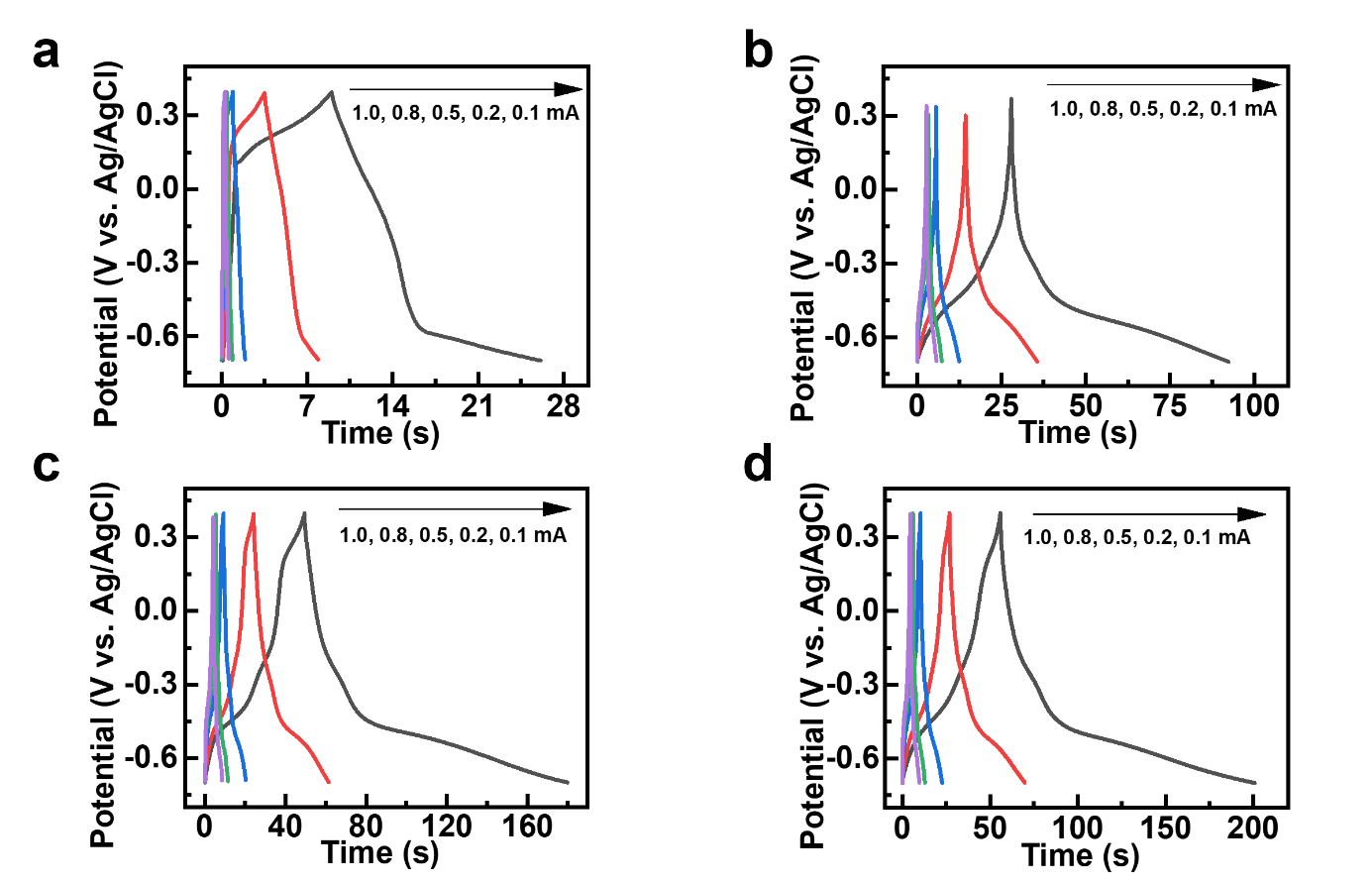


**Figure S6.** The charge-discharge curves of (a) the Au NPs@PANI nanofilm, (b) the W_18_O_49_ NWs film, (c) the Au NPs @PANI/W_18_O_49_NWs composite nanofilm and (d) the W_18_O_49_NWs/Au NPs @PANI composite nanofilm at current densities of 1.0, 0.8, 0.5, 0.2, and 0.1 mA/cm^2^.


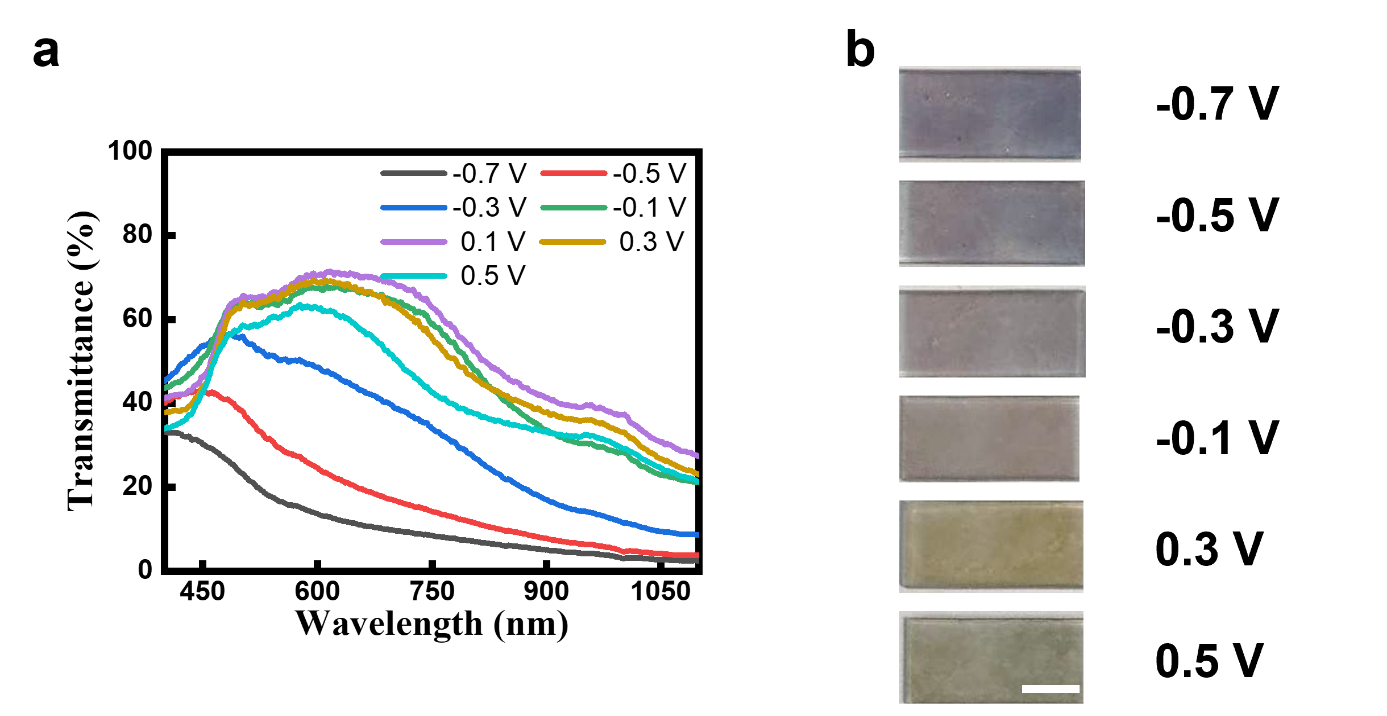


**Figure S7.** (a) The UV*-vis* transmittance spectra of Au NPs@PAN/W_18_O_49_ NWs composite nanofilm. (b) Optical picture of Au NPs@PAN/W_18_O_49_ NWs composite nanofilm in different bias voltages. Scale bar: 0.5 cm.


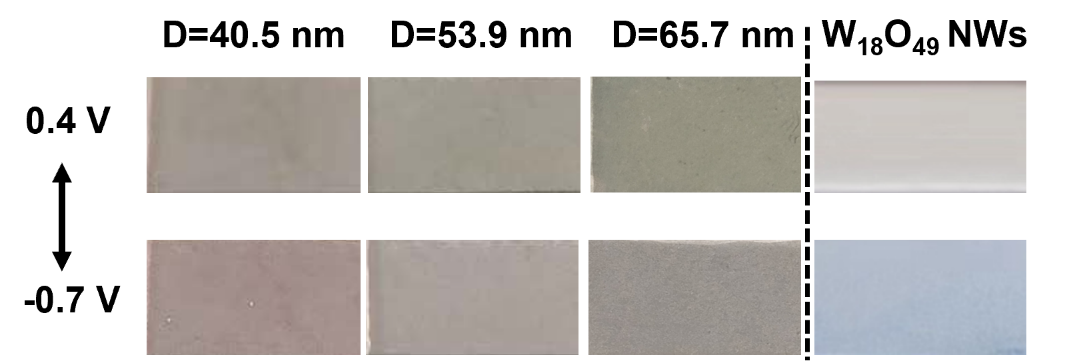


**Figure S8.** Optical images of AuNPs@PANI Film (polymerized 1 to 3 times) and W_18_O_49_ NWs on one ITO-glass electrode at different bias voltage.


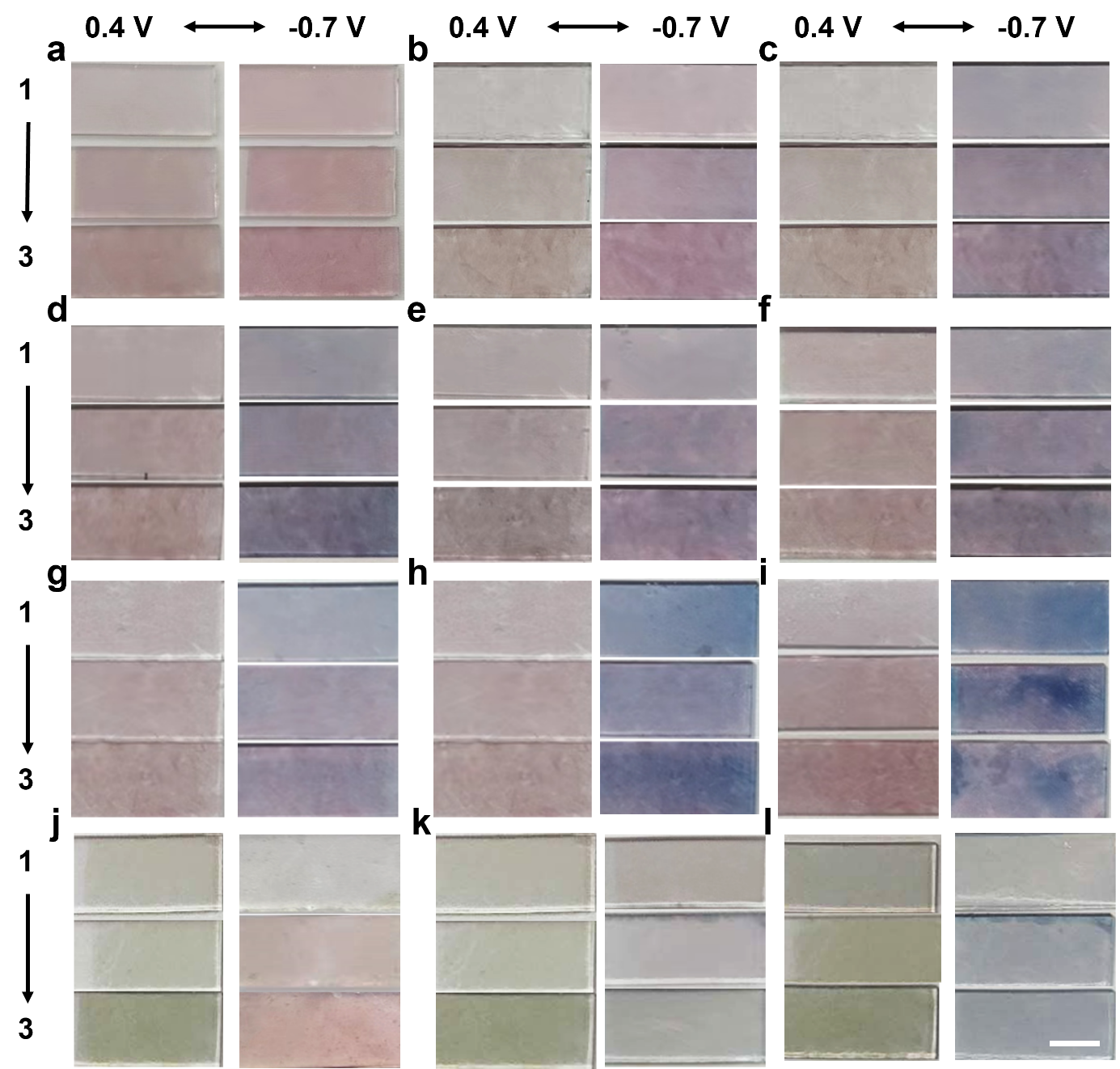


**Figure S9.** (a-i) 1-3 layers of Au NPs@PANI-1 nanofilm with 0, 11.6, 21.9, 38.3, 48.2, 66.4, 95.0, 117.5, 145.3 nm of W_18_O_49_ NWs. (j-l) 1-3 layers of Au NPs@PANI-3 nanofilm with 0, 11.6, 21.9 nm of W_18_O_49_ NWs. Scale bar: 0.5 cm.


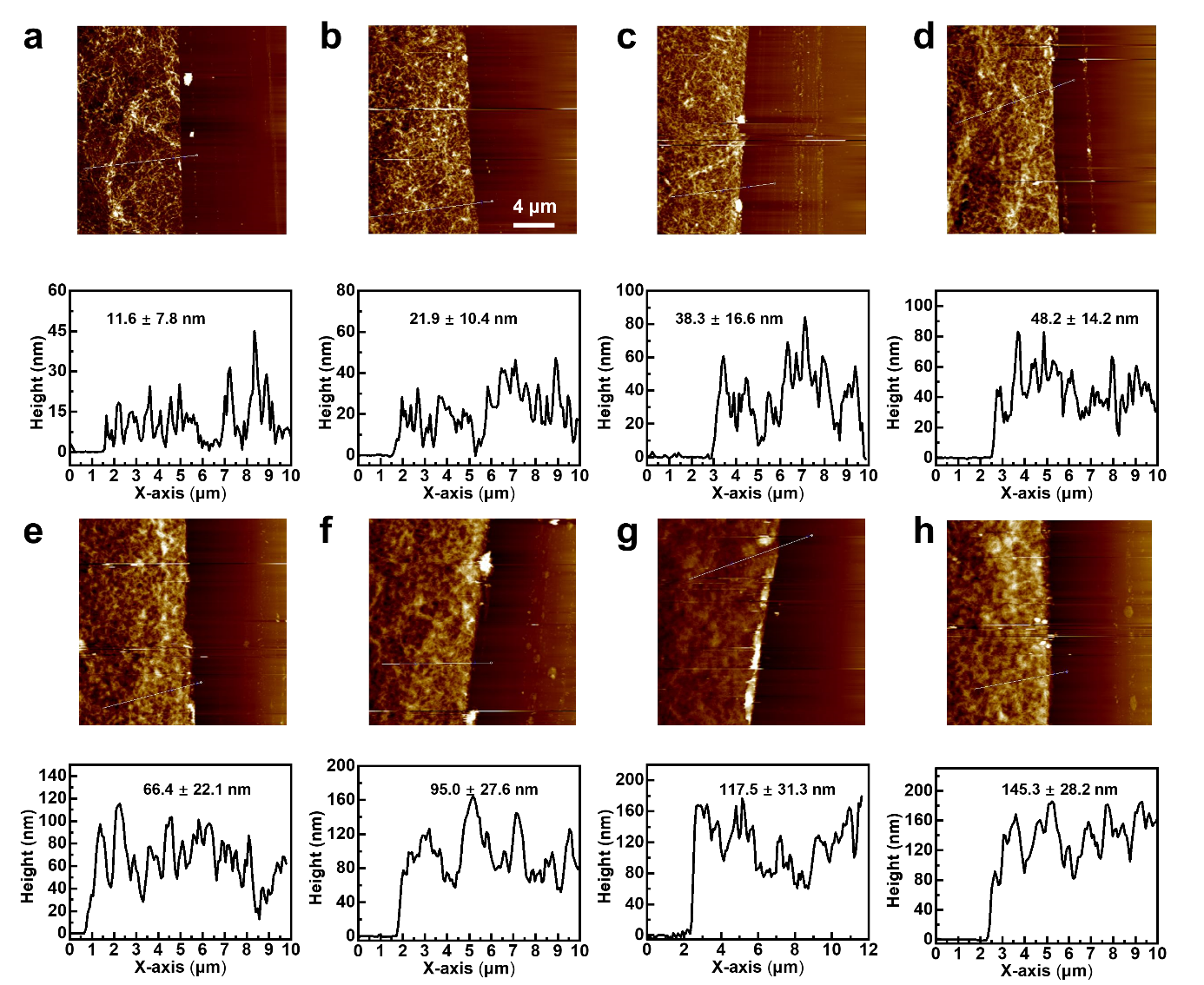


**Figure S10.** (a-h) The height of the film prepared by W_18_O_49_ NW layer in different thickness (from 11.6 ± 7.8 nm to 145.3 ± 28.2 nm).


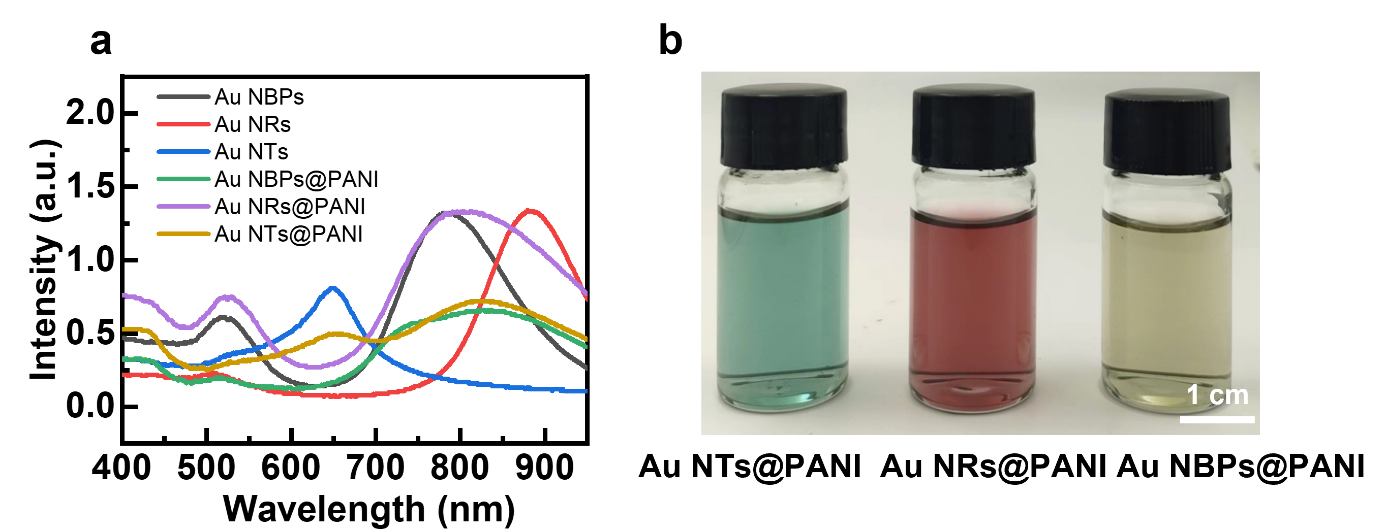


**Figure S11.** (a) The UV*-vis* absorption spectra of Au NTs, Au NRs, Au NBPs, Au NTs@PANI, Au NRs@PANI, and Au NBPs@PANI core/shell nanostructures. (b) The optical photographs of Au NTs@PANI, Au NRs@PANI, and Au NBPs@PANI solution.


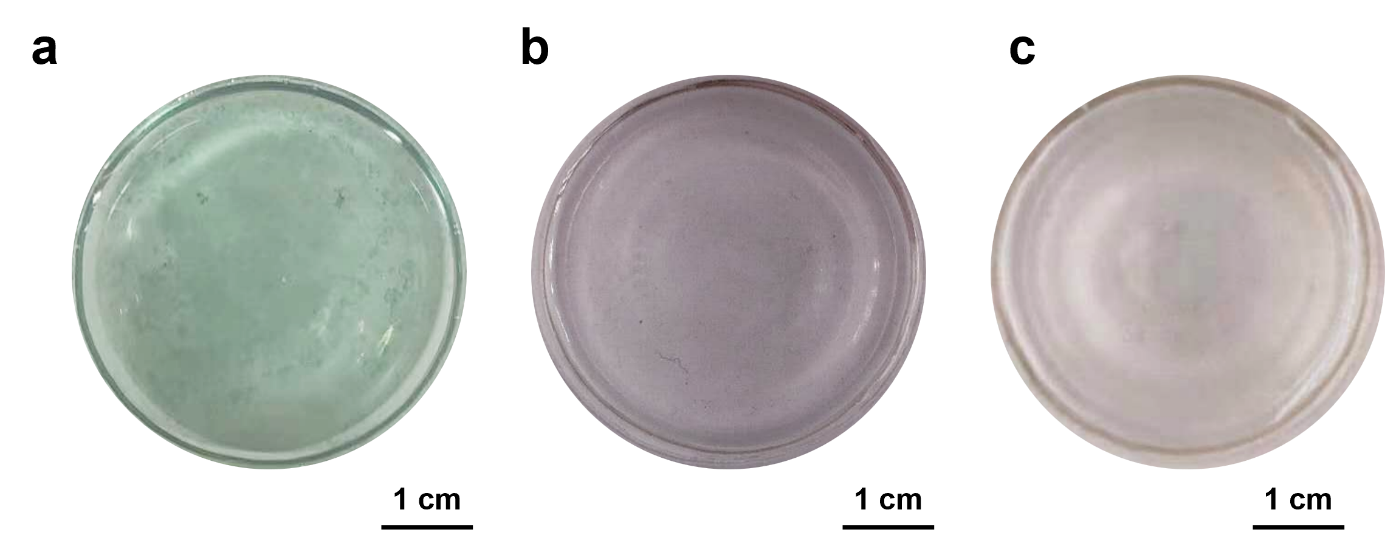


**Figure S12.** The optical photographs of Au NTs@PANI (a), Au NRs@PANI (b), Au NBPs@PANI (c) monolayer film after assembled at water-hexane interface.


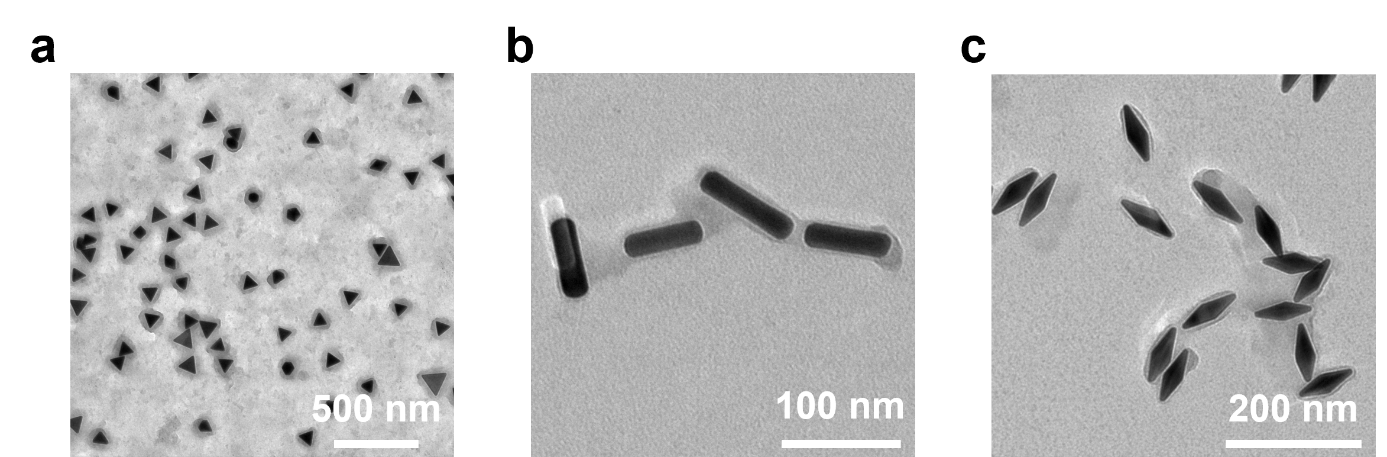


**Figure S13.** TEM images of Au NTs@PANI (a), Au NRs@PANI (b), Au NBPs@PANI (c).


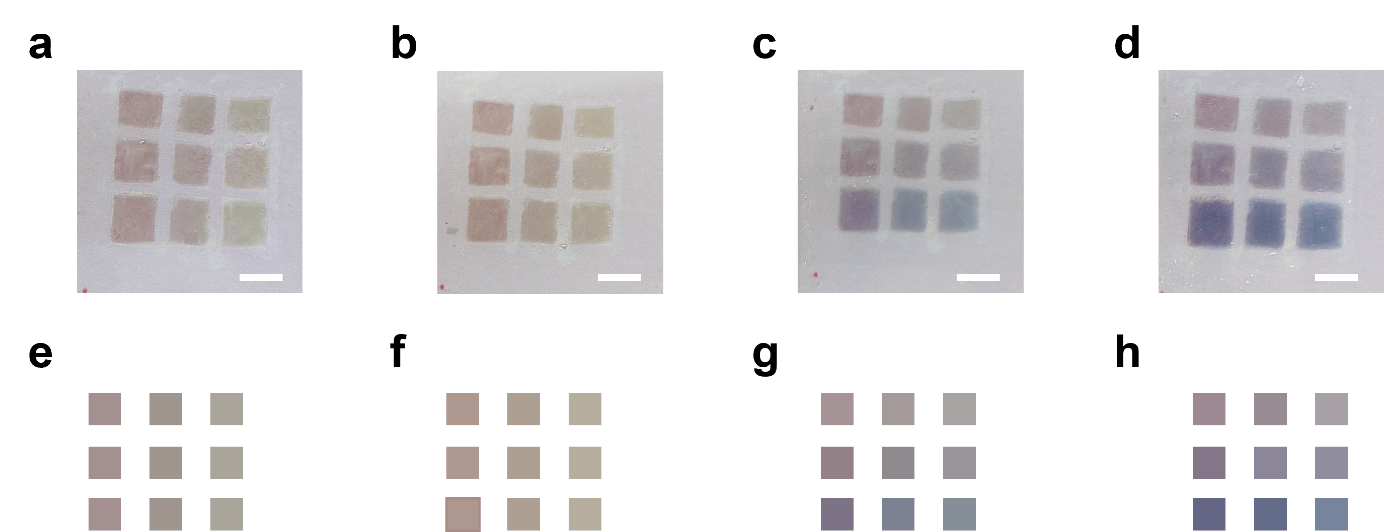


**Figure S14.** (a-d) Optical pictures of nine-cell grid composed of different combinations of Au NPs@PAN/W_18_O_49_ NWs composite nanofilm with the voltage changes from 0.4 V, -0.1 V, -0.4 V and -0.7 V. (e-h) Simulated picture of nine-cell grid composed of different combinations of Au NPs@PAN/W_18_O_49_ NWs composite nanofilm with the voltage changes from 0.4 V, -0.1 V, -0.4 V and -0.7 V. Scale bar: 0.5 cm.


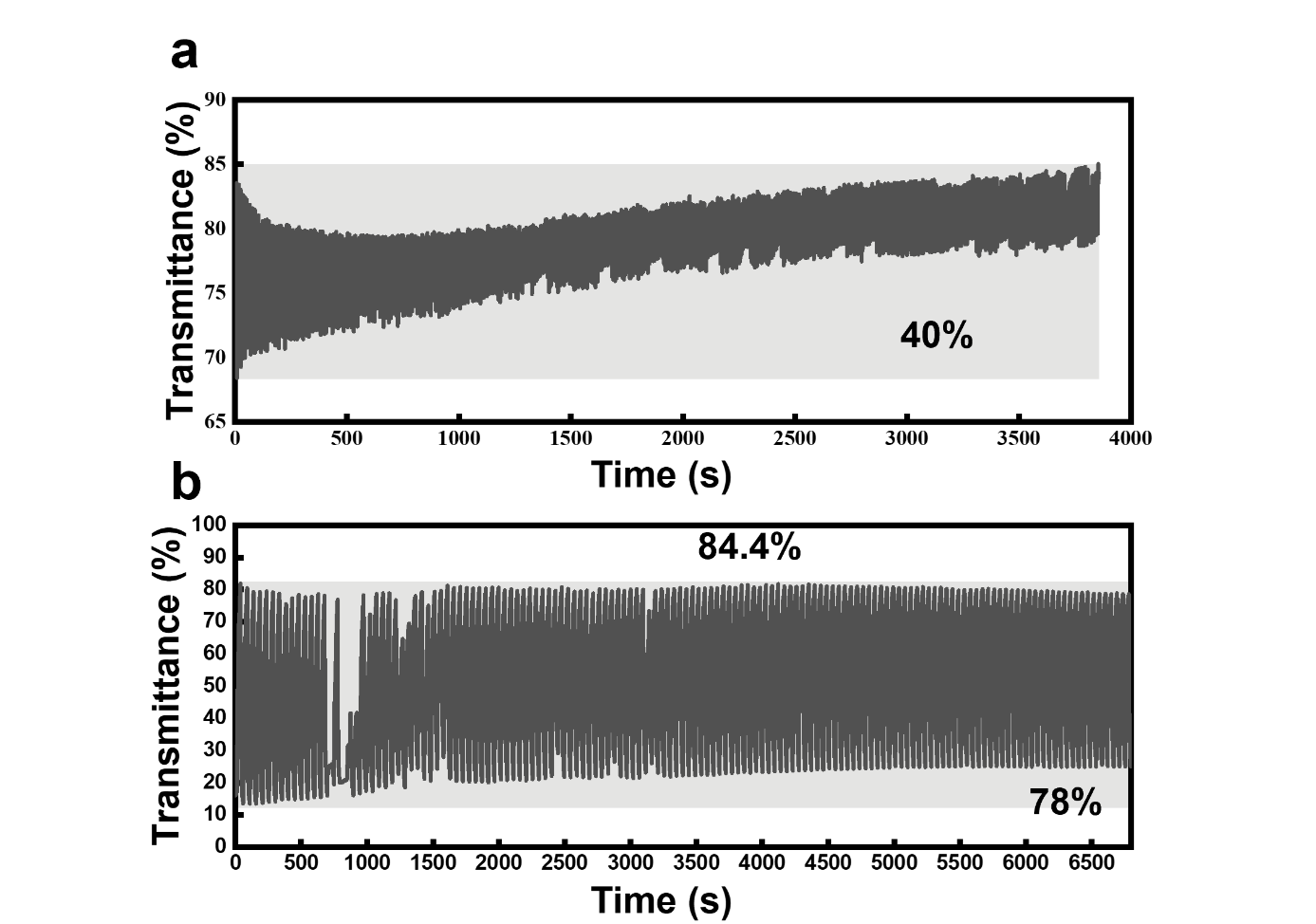


**Figure S15.** (a) Electrochromic switching behaviors of the as-prepared Au NPs@PANI nanofilm monitored at 600 nm at a voltage of -0.7 V for 5 s and 0.4 V for 5 s for cycles. (b) Electrochromic switching behaviors of the as-prepared W_18_O_49_ NWs nanofilm monitored at 600 nm at a voltage of -0.7 V for 30 s and 0.4 V for 30 s for cycles.


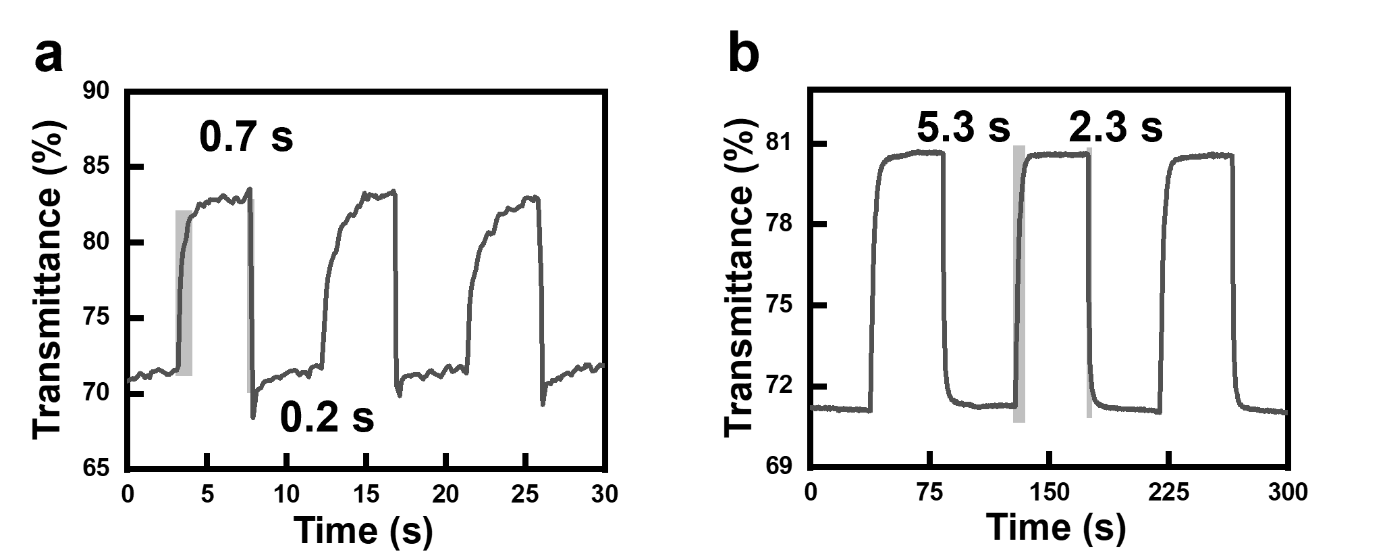


**Figure S16.** (a) Electrochromic switching behaviors of the as-prepared Au NPs@PANI nanofilm monitored at 600 nm at a voltage of -0.7 V for 5 s and 0.4 V for 5 s for cycles. (b)Electrochromic switching behaviors of the as-prepared W_18_O_49_ NWs nanofilm monitored at 600 nm at a voltage of -0.7 V for 40 s and 0.4 V for 40 s for cycles.


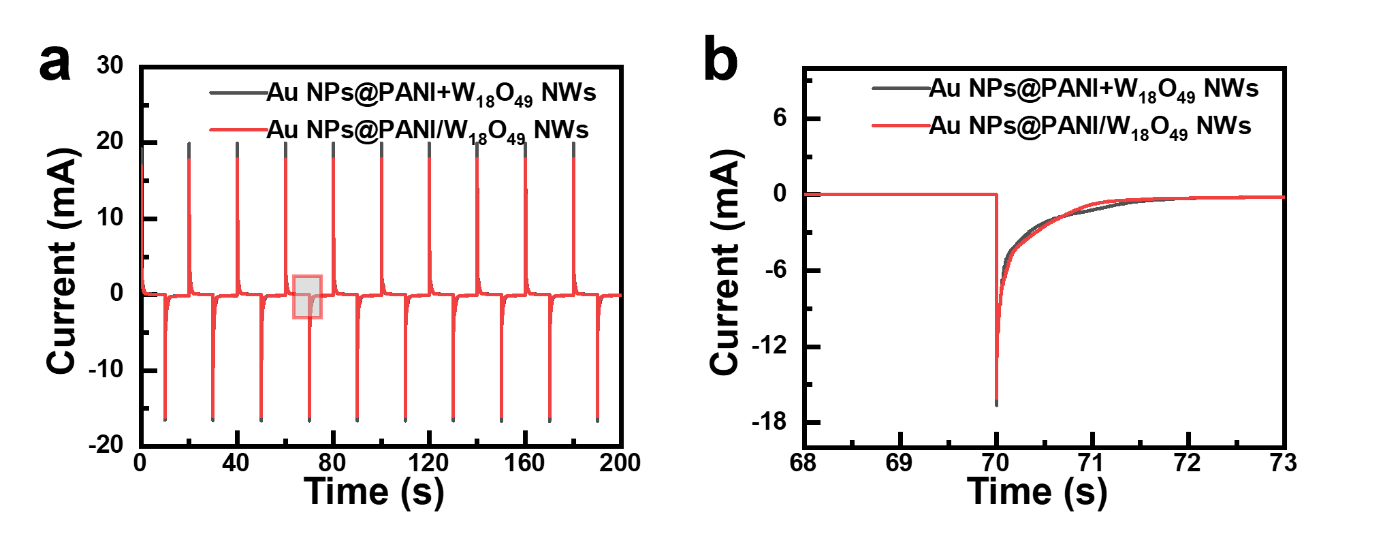


**Figure S17.** (a) Chronoamperometry of the Au NPs@PANI/W_18_O_49_ NWs composite nanofilm and Au NPs@PANI film+W_18_O_49_ NWs film (b) is a partial magnification of (a).


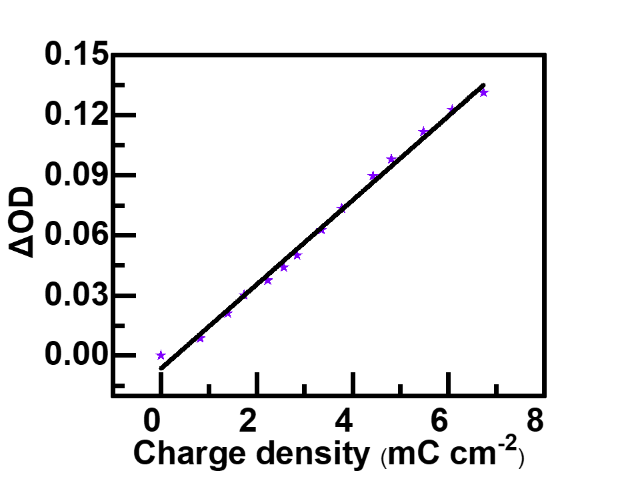


**Figure S18.** Plots of the optical density (ΔOD) as a function of charge density (ΔQ) of Au NPs@PANI/W_18_O_49_ NWs composite film, which was derived from the electrochromic states of -0.7 to 0.4 V.


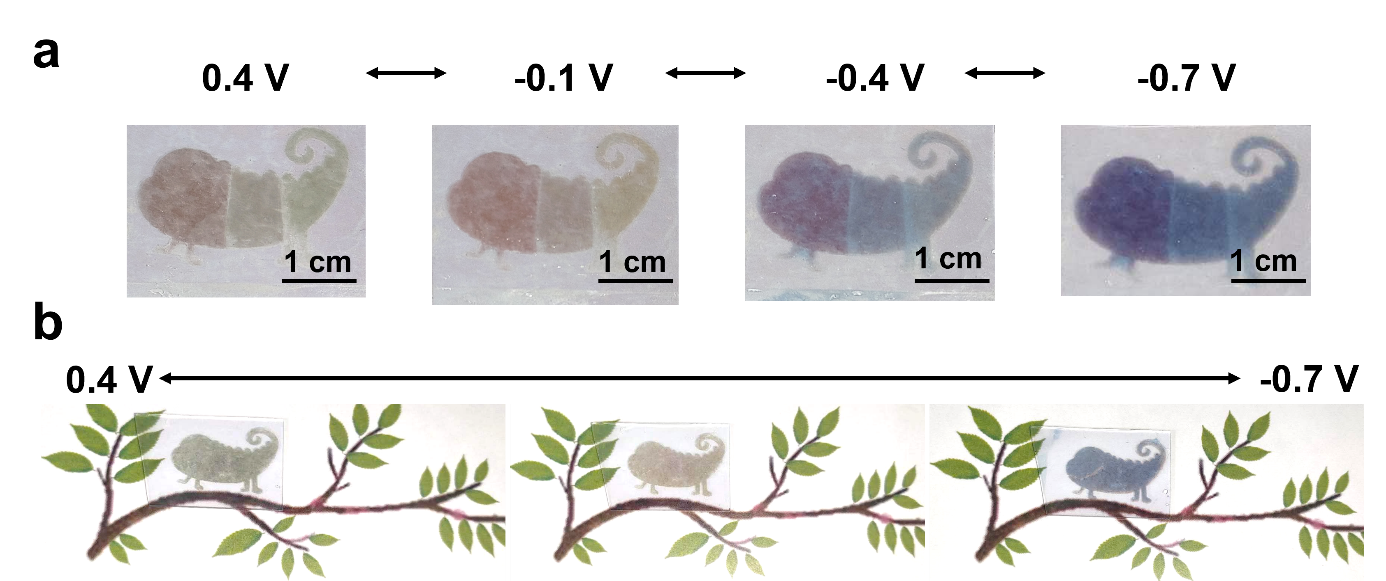


**Figure S19.** (a) Chameleon fabricated based on various combination of Au NPs@PANI/W_18_O_49_ NWs. (b) Chameleon camouflage itself on branches.


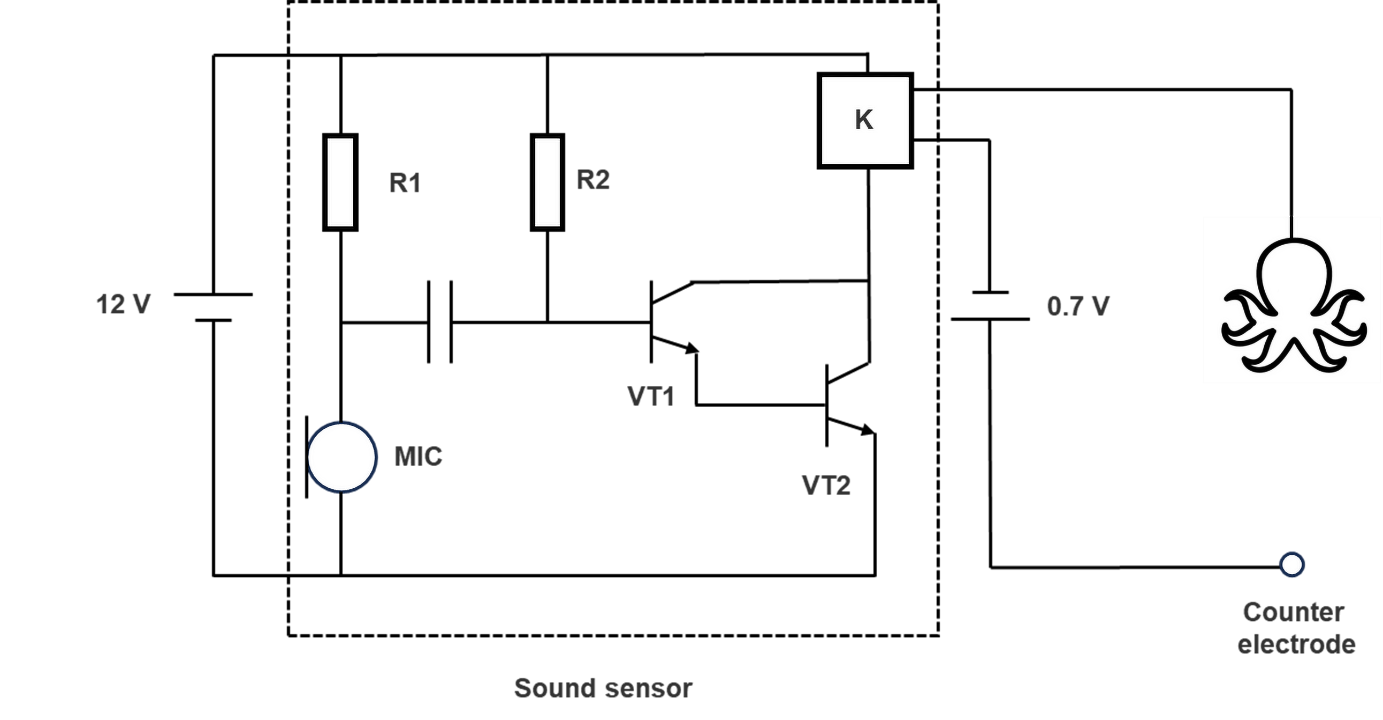


**Figure S20.** Once the sound signals reach a predetermined threshold, the sensor automatically triggers the connection of the circuit.
